# Extending evidence on food insecurity and ultra-processed food consumption: insights from the Philippine food system

**DOI:** 10.1017/S1368980026102730

**Published:** 2026-05-21

**Authors:** Kervin B. Untalan, Genessa Jesy T. Pagote, Kyle Renz G. Tejero

**Affiliations:** 1 Food Technology Department, Bukidnon State University, Philippines; 2 Natural Science Department, College of Arts and Sciences, Bukidnon State University, Philippines

**Keywords:** Food insecurity, Ultra-processed foods, Food environments, Public policy

Dear Editor,

The recent article by Fulay *et al.* ^(1)^ examined the associations between food insecurity, Supplemental Nutrition Assistance Program (SNAP) participation and ultra-processed food (UPF) acquisitions among US households. The study demonstrated that while SNAP participation alone was not significantly associated with UPF purchases, marginal food insecurity was linked to higher acquisition of UPFs, underscoring the structural role of food insecurity in shaping dietary patterns beyond programme participation.

Extending these findings to low- and middle-income countries (LMICs) such as the Philippines provides important contextual insights. Unlike the USA, where SNAP offers structured and regulated food purchasing support, food assistance in the Philippines, largely institutionalised through Republic Act No. 11310 (4Ps), remains fragmented and only partially nutrition-sensitive^(2)^. In this context, dietary outcomes are shaped less by programme participation and more by broader food environments, suggesting that pathways linking food insecurity and UPF consumption differ across settings.

The Philippines is experiencing a ‘double burden of malnutrition’, where undernutrition coexists with rising overweight and obesity^(3)^. As described by Monteiro *et al.*,^(4)^ UPFs are increasingly displacing traditional diets due to affordability, convenience and palatability. Baker *et al.* ^(5)^ further highlight that in LMICs, the expansion of UPFs is closely tied to economic constraints and food system transformations. These dynamics suggest that the associations observed by Fulay *et al.* ^(1)^ may be amplified where access to fresh and minimally processed foods is structurally limited.

A key dimension in the Philippine context is the role of informal food systems. Reardon and Timmer^(6)^ note that small-scale and informal retailers dominate food distribution in many LMICs. In the Philippines, sari-sari stores and street vendors serve as primary food access points, particularly for low-income households, and are often characterised by a high availability of ultra-processed, shelf-stable products. Consequently, dietary practices are shaped less by individual agency and more by constrained food environments.

Beyond physical access, these dynamics may also be understood through a communication lens. Food environments function as systems of both distribution and influence, where marketing, product visibility and informal knowledge shape dietary practices. As emphasised by the WHO^(7)^, pervasive marketing of foods high in fats, sugars and salt significantly influences dietary preferences, particularly among vulnerable populations. In many LMIC settings, access to reliable and culturally appropriate nutrition information remains uneven, while commercial promotion of UPFs is widespread. Thus, food insecurity is not only a material constraint but also a communicative one, where unequal access to information mediates dietary choices.

These patterns are further shaped by regulatory contexts. The WHO^(7)^ calls for comprehensive government-led restrictions on unhealthy food marketing. This aligns with the High Level Panel of Experts^(8)^ framing of food systems as structured by socio-economic and policy conditions. Hawkes *et al.* ^(9)^ advocate for ‘double-duty actions’ addressing shared drivers of undernutrition and diet-related non-communicable diseases, while Popkin *et al.* ^(10)^ highlight how global dietary shifts towards UPFs contribute to the accelerating double burden of malnutrition.

Taken together, the findings of Fulay *et al.*,^(1)^ when considered in the Philippine context, suggest that food insecurity and UPF consumption are embedded within broader structural constraints, including informal food systems, limited regulation, inequitable food access and communication inequalities. Addressing these challenges requires moving beyond food assistance programmes towards integrated food system approaches that strengthen local food environments, regulate food marketing and improve access to reliable nutrition information.

In conclusion, the study by Fulay *et al.* ^(1)^ provides an important empirical foundation for understanding the relationship between food insecurity and UPF acquisition. Extending this evidence to LMIC settings highlights the need for context-specific, systems-level interventions addressing both material and communicative drivers of dietary change.
